# Workplace demands, resources, and well‐being among police staff working in forensic services

**DOI:** 10.1111/1556-4029.70179

**Published:** 2025-09-08

**Authors:** Jacob J. Keech, Jacqueline M. Drew

**Affiliations:** ^1^ School of Applied Psychology Griffith University Nathan Queensland Australia; ^2^ School of Criminology & Criminal Justice and Griffith Criminology Institute Griffith University Nathan Queensland Australia

**Keywords:** burnout, crime scene investigator, crime scene technicians, CSI, law enforcement, police; occupational stress, vicarious trauma

## Abstract

Forensic staff play a crucial role in law enforcement through providing specialist services to police agencies in criminal investigations. Given the unique work, including frequent exposure to potentially distressing material, administrative workloads, and other work‐related pressures, forensic staff are at risk of increased occupational stress. The current study examined the demands and resources associated with stress‐related outcomes among forensic staff. It further provides descriptions of the coping strategies used, perceptions of organizational support resources, and attitudes toward help‐seeking and using sick leave. Participants were 114 sworn and non‐sworn forensic staff working in an Australian law enforcement organization. The study used a mixed methods design with participants completing survey questions online. Quantitative data were analyzed using bivariate correlations and partial least squares regression analyses. Qualitative data were analyzed using thematic analysis. Results identified the key role of occupational and organizational stressors, and forensic‐specific job‐related demands, in predicting various stress‐related outcomes. Supervisor support, peer support, and psychosocial safety climate also had a key role in predicting stress‐related outcomes among forensic staff. Law enforcement organizations employing staff in forensic job roles should take a holistic approach to optimizing demands which not only focuses on trauma, but also on mitigating occupational and organizational stressors. Demands specific to the role of forensics also need to be considered. In an effort to offset job demands, police agencies should seek to uplift the capacity of key resources such as supervisors and peers and should focus on ensuring a positive psychosocial safety climate.

**Highlights**

- Forensic staff face unique stressors beyond trauma exposure in their daily work.
- Organizational and occupational demands strongly predict stress‐related outcomes.
- Supervisor and peer support are key resources for managing forensic staff stress.
- Positive psychosocial safety climate is associated with lower stress in forensic workplaces.
- Findings support holistic stress mitigation strategies tailored to forensic roles.

## 1 INTRODUCTION

Experiencing stress at work, commonly referred to as occupational stress, has the potential to negatively impact the health, well‐being, and performance of employees [1, 2, 3]. It is likely that individuals working in high‐demand environments will be at increased risk of work‐related stress. Work‐related stress is inextricably linked to negative outcomes such as psychological distress and burnout [4]. Research has established that those working in law enforcement, regardless of their role, are likely to experience high levels of work‐related stress, and this stress is a key factor in the high rates of psychological ill‐health that are reported for this employee cohort [5, 6, 7, 8, 9]. More specifically, there are particular groups within the law enforcement community that may be at even higher risk. One group that is often identified is forensic staff [10, 11], due to the unique demands they face. As such, it is critical to better understand which work‐related stressors are most strongly associated with important outcomes such as burnout, psychological distress, and vicarious trauma, and also to identify resources and strategies that can be put in place to better support their personal and professional well‐being.

### 1.1 Occupational stress

Occupational stress is considered a major threat to the health and well‐being of workers. It can create challenges for organizations due to its association with outcomes such as employee burnout, turnover, inefficiency, and absenteeism [1, 2, 3]. Occupational stress refers to the experience of physical and emotional strain in response to work‐related demands that surpass a worker's resources and ability to cope [1, 12, 13]. Workplace stress has significant economic implications for workplaces. It has been estimated that job stress related to depression stemming from excessive workplace pressures costs the Australian economy $730 million per year [14].

As discussed below, workplace stress has become a particular concern in law enforcement environments due to the alarming prevalence rates of adverse well‐being outcomes that result from their unique work experiences. Much discussion has focused on posttraumatic stress [15]. While rates vary, Papazoglou and Andersen [16] found that 35% of the officers that they surveyed experience post‐traumatic stress symptoms. Depression and anxiety disorders are also common [17, 18].

### 1.2 Job demands–resources model

**FIGURE 1:**
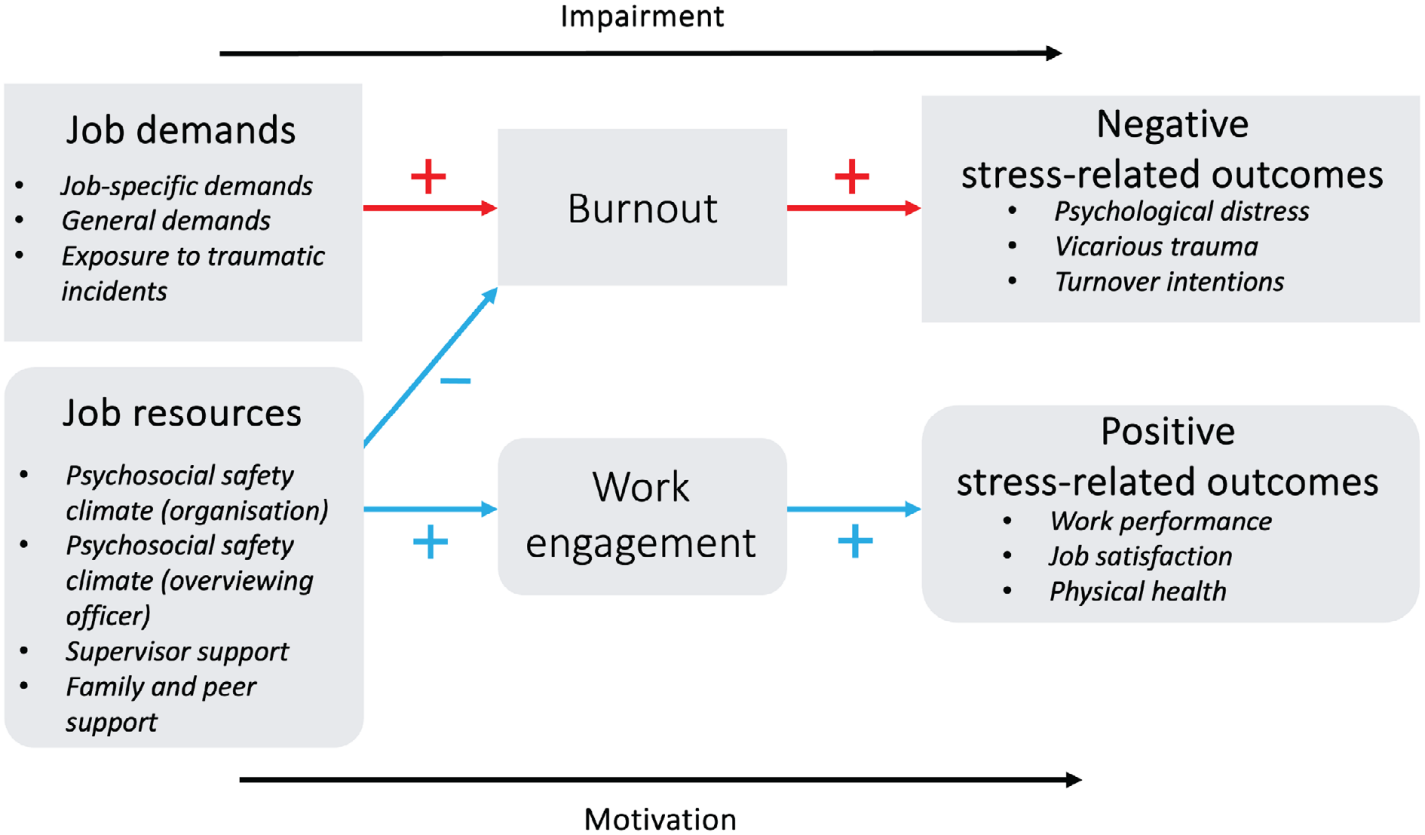
The job demands–resources model adapted for the current context based on Schaufeli [21]. The figure depicts variables investigated in the current study based on their role within the job demands–resources model.

An often‐used approach for understanding *stress‐related outcomes* in the workplace, whether they be negative (including psychological distress and vicarious trauma) or positive (including work performance and job satisfaction), is the job demands–resources (JD‐R) model (see Figure 1). It is an approach that can be used to understand how job demands and job resources are associated with outcomes, such as burnout and work engagement [4]. *Burnout* is a psychological syndrome that is characterized by emotional, mental, and physical exhaustion and emerges in response to employees experiencing chronic stress in the workplace [19]. *Work engagement* is a work‐related state of mind that includes positivity and fulfillment and is characterized by vigor, dedication, and absorption [20]. Employees who experience high *job demands* (e.g., high workload, conflict, stress) and a lack of resources in their work are vulnerable to burnout, which in turn may lead to diminished motivation, performance, and health. In contrast, when *job resources* (e.g., social support from peers, family, and supervisors; positive psychosocial safety climate) are high, employees tend to experience positive outcomes such as increased motivation and engagement—even in demanding jobs [21]. *Psychosocial safety climate* is an organizational resource comprised of policies, procedures, and practices that foster the psychological health and safety of employees [22]. The JD‐R model was used to guide the current research. It provides a useful framework to identify aspects of the workplace that should be examined to provide insights into the connection between specific workplace factors and workplace engagement and well‐being among forensic staff. The JD‐R model points to the importance of creating high‐resource work environments, particularly in job roles such as forensic services work, which may involve high levels of mental and physical demands.

### 1.3 Stress and well‐being in forensic staff working in law enforcement

Research on occupational stress has demonstrated that employees working in policing or law enforcement are at a higher risk of experiencing job‐related stress, burnout, and mental health conditions when compared to the general population [5, 9, 23, 24]. A recent large‐scale survey of over 8000 Australian police service employees revealed substantial rates of low well‐being (32%), high to very high distress (31%), and the presence of at least one mental health diagnosis (23%) among respondents [25]. The demands of working in policing are multifaceted, and research suggests that, even after controlling for exposure to traumatic incidents (i.e., *trauma stress*), organizational and operational stressors play a key role in determining occupational stress in policing [7, 26]. *Organizational stressors* are those job demands that are associated with wider organizational aspects, such as workplace procedures and culture [27, 28]. Examples of organizational stressors include unequal sharing of work responsibilities, bureaucratic red tape, and excessive administrative duties [26]. Conversely, *operational stressors* are those job demands that are associated with the inherent aspects of working in a specific occupation [27, 28]. Examples of operational stressors include working alone at night, risk of being injured on the job, and shift work [26]. While these stressors may commonly occur across policing contexts, some specialist groups may also experience a range of stressors unique to their specific type of work in policing.

Representing one specialist group within law enforcement, forensic staff work alongside other police officers in criminal investigations. They typically provide a range of services, both at the scene of crimes and in laboratory contexts. These services may include undertaking forensic examinations at active crime scenes, collecting and examining exhibits, and providing evidence and specialist testimony in court. Forensic staff face unique job demands such as more frequent exposure to graphic details of crimes, tragedies, and human suffering compared to many of their law enforcement colleagues [10]. Limited existing research indicates that continuously dealing with these demands can negatively impact the health and well‐being of forensic staff [29]. Given these specific challenges, a small but growing number of studies are investigating the effects of working in forensic contexts on physical and psychological health. Some research has begun to examine the role of coping behaviors and job attitudes in buffering these effects.

### 1.4 Common stressors in forensic staff

Stemming from the type of work performed by forensic staff, previous research has found that common *forensic staff‐specific job demands* include examining active scenes, shift work, excessive administrative workload, limited promotability, and lacking a defensive weapon [30, 31, 32]. Experiencing job‐related stress in these contexts has been associated with increased heart rate activity during scene examinations [33], anxiety and psychological distress [10], indicators of post‐traumatic stress disorder [34, 35], as well as hypervigilance, stress exhaustion, burnout, and depression [31]. Limited research has explored how stress‐related outcomes stemming from forensics work might be reduced through protective factors (i.e., *job and personal resources*). This may include protective support resources provided by the organization and individual protective factors, such as the use of effective coping strategies [10, 31].

### 1.5 Coping strategies used by forensic staff

Forensic staff dealing with highly stressful work environments may use a variety of coping strategies which vary in effectiveness. For instance, one study found that avoidance coping (e.g., denial, distraction, seeking alternative rewards) was the most frequently used strategy among a sample of crime scene technicians [36]. However, it was found to be least effective when coping with traumatic work‐related experiences and was associated with increased physical health problems. Other coping behaviors that may reduce stress in the short term include behavioral disengagement, self‐blame, or substance use [10]. The effectiveness of such coping strategies in the longer term remains unclear.

Adaptive coping strategies that have been observed in research among forensic staff include using humor, engaging in peer debriefing and active coping (e.g., seeking social support), using positive reframing, practicing acceptance, and mentally preparing for demanding crime scenes [10, 30, 32, 35]. Indeed, the use of adaptive strategies has been associated with positive outcomes for forensic staff, such as greater work engagement, well‐being, and longevity [10, 30]. Therefore, coping skills appear to be an important factor in increasing well‐being outcomes when working in highly demanding occupations, such as forensic services.

### 1.6 The current study

While some studies have focused on identifying the type and prevalence of stress, stress‐related outcomes, and coping strategies used by forensic staff, further research is needed to adequately understand how job demands predict stress and well‐being of this cohort. Despite the high frequency of exposure to traumatic situations, little research has examined stress and related outcomes among forensic and crime scene personnel [10, 36]. Therefore, the current study aimed to examine the factors associated with stress‐related outcomes among forensic staff working in law enforcement. The current study also aimed to identify the protective resources that can effectively improve health, work engagement, and performance of forensic staff. Finally, the current study aimed to examine forensic staff descriptions of the coping strategies they use, their perceptions of support resources provided by their organization, and their attitudes toward help‐seeking and using sick leave. The study involved six specific research questions, which are outlined below.

RQ1: Which background and demographic characteristics are associated with stress‐related outcomes among forensic staff?
RQ2: Which general demands predict stress‐related outcomes among forensic staff?
RQ3: Which forensic‐specific demands predict stress‐related outcomes among forensic staff?
RQ4: Which resources predict stress‐related outcomes among forensic staff?
RQ5: What coping strategies do forensic staff describe using to manage their demands?
RQ5: What support resources do forensic staff describe as being provided by their organization?
RQ6: What are the attitudes of forensic staff toward help‐seeking and using sick leave?

## 2 MATERIALS AND METHODS

### 2.1 Participants

Participants were 114 staff from the Forensic Services Group and Forensic Crash Unit within an Australian state police organization. Throughout this article, the term forensic staff has been used to refer to both police and civilian staff members employed within these operational groups. The sample represents a response rate of 19.2% of the total 594 members across both groups. Of the respondents, almost 86% were sworn police and 14% were civilian police staff. Almost half of all respondents held the rank of constable or senior constable (49.1%), followed by sergeants (29.8%), and 7.1% held the rank of inspector or higher. Civilian police staff were employed at AO3 (6.1%), AO4 (2.6%), and AO5 (1.8%) levels (general through to managerial administrative officer classifications). The majority of the sample were based in police districts throughout the state (76%), and 24% worked in police headquarters. In respect to specific job role, 51.8% worked in Scenes of Crime; the next largest group worked in the Forensic Crash Unit (12.3%), followed by the Fingerprint Bureau (11.4%). An almost even split of male and female staff responded to the survey. The largest group of staff (44%) had 21 or more years of service with the organization. Most staff (68%) reported having 10 years or more of service in forensic services. Please see Table S1 for detailed sample characteristics.

### 2.2 Design and procedure

The study was approved by the Human Research Ethics Committee at two universities (reference: 2021/793 and A221725) and by the Research Committee of the police organization involved in the study. The study adopted a cross‐sectional correlational design. Data for the current research were collected as part of a larger, Australian Research Council (ARC) Linkage (LP200200834) funded project. This project involved a state‐wide survey sent to all personnel within the police organization. The data were collected using an online anonymous survey administered through the Lime Survey online platform. The link to the survey was distributed via an internal organizational email to the workforce by the Commissioner of Police. Survey promotion was also undertaken through distribution via emails by the two police unions who service the police agency. Reminder emails were sent to the whole workforce, and specific reminder emails were sent to the Forensic Services Group by the Superintendent in charge of the group. Data were collected between March 20, 2022 and May 1, 2022.

### 2.3 Measures

A battery of validated scales, scales constructed for the current research, and open‐ended questions were administered to participants via the online survey. All scales exhibited good reliability. Alpha reliability coefficients are reported in Table S2.

#### 2.3.1 General demands

##### Operational stress

Operational stress was measured using an adapted version of the 20‐item Operational Police Stress Questionnaire [28] (PSQ‐Op). Participants responded on 8‐point Likert scales (1 = *have not experienced* to 8 = *a lot of stress*), with higher scores indicating greater experience of stress arising from job demands that are associated with are associated with the inherent aspects of working in the specific occupation [28].

##### Organizational stress

Organizational stress was measured using 22 items which were an adapted version of the 22‐item Organizational Police Stress Questionnaire [28] (PSQ‐Org). Participants responded on 8‐point Likert scales (1 = *have not experienced* to 8 = *a lot of stress*), with higher scores indicating greater experience of stress arising from job demands that are associated with wider organizational aspects, such as workplace procedures and culture [28].

##### Trauma stress

Trauma stress was measured using 19 items which were an adapted version of the Critical Incident History Questionnaire [37] adapted for the current study. Participants responded on 8‐point Likert scales (1 = *have not experienced* to 8 = *a lot of stress*) with higher scores indicating greater experience of stress arising from exposure to traumatic incidents.

#### 2.3.2 Forensic staff specific demands

Forensic staff specific demands were measured using a 13‐item scale constructed for the current research. The scale was constructed based on stressors originally identified in qualitative research by Sollie and colleagues [32] and revised based on feedback from forensic staff employed in the agency involved in the current study. Participants responded on 5‐point Likert scales (1 = *never/hardly ever* to 5 = *always*) with higher scores indicating greater experience of the demand specified.

#### 2.3.3 Resources

##### Psychosocial safety climate (organization)

Psychosocial safety climate at the organization level was measured using the 4‐item Psychosocial Safety Climate Scale (PSC‐4) [38]. Participants responded on 7‐point Likert scales (1 = *very strongly disagree* to 7 = *very strongly agree*) with higher scores indicating higher perceptions of psychosocial safety climate (organization), which is the extent to which an employee believes that the organization and senior management have policies, procedures, and practices in place that foster the psychological health and safety of employees [22].

##### Psychosocial safety climate (overviewing officer)

Psychosocial safety climate at the overviewing officer level was measured using the 3‐item adapted version of the Psychosocial Safety Climate scale (PSC‐4) [38]. Participants responded on 7‐point Likert scales (1 = *very strongly disagree* to 7 = *very strongly agree*) with higher scores indicating higher perceptions of psychosocial safety climate (overviewing officer), which is the extent to which an employee believes that their overviewing officer promotes a climate in which their psychological safety needs are met [39]. The overviewing officer was described to participants as referring to the officer in charge of their position, which is usually the person their direct supervisor reports to.

##### Supervisor support

Supervisor support (direct supervisor) was measured using the 4‐item UK social support scale referenced to participants' direct supervisor [40]. Participants responded on 7‐point Likert scales (1 = *very strongly disagree* to 7 = *very strongly agree*) with higher scores indicating a greater perception that their work‐related support needs are being met by their direct supervisor [39].

##### Peer support

Peer support was measured using the 3‐item UK social support scale for peer social support [40]. Participants responded on 7‐point Likert scales (1 = *very strongly disagree* to 7 = *very strongly agree*) with higher scores indicating a greater perception that their personal support needs are being met by their peers [39].

##### Family support

Family support was measured using the 2‐item UK social support scale for family support [40]. Participants responded on 7‐point Likert scales (1 = *very strongly disagree* to 7 = *very strongly agree*) with higher scores indicating a greater perception that their personal support needs are being met by their family members [39].

#### 2.3.4 Stress‐related outcomes

##### Burnout (exhaustion)

Burnout was measured using the 8‐item exhaustion subscale of the Oldenburg Burnout Inventory (OLBI) [41]. Participants responded on 7‐point Likert scales (1 = *very strongly disagree* to 7 = *very strongly agree*) with higher scores reflecting greater exhaustion, which is a psychological syndrome that is characterized by emotional, mental, and physical exhaustion and emerges in response to employees experiencing chronic stress in the workplace [19].

##### Psychological distress

Psychological distress was measured using the 10‐item Kessler Psychological Distress Scale (K‐10) [42]. Participants responded on 5‐point Likert scales (1 = *none of the time* to 5 = *all of the time*) about how they have felt during the previous 4 weeks. Higher scores indicated greater psychological distress, which is a state of emotional suffering that is usually marked by symptoms of anxiety and depression and is associated with difficulties in coping with stressors and demands [43].

##### Vicarious trauma

Vicarious trauma was measured using the six‐item Vicarious Trauma Scale [44]. Participants responded on 7‐point Likert scales (1 = *very strongly disagree* to 7 = *very strongly agree*) with higher scores indicating a greater experience of vicarious trauma, which is a persistent emotional response to ongoing exposure to the trauma experience of others [45].

##### Work engagement

Work engagement was measured using the two items from the Oldenburg Burnout Inventory [41] as reported by Dollard and Bakker [22]. Participants responded on 7‐point Likert scales (1 = *very strongly disagree* to 7 = *very strongly agree*) with higher scores reflecting greater work engagement, which is a work‐related state of mind that is characterized by positivity and fulfillment [20].

##### Work performance

Work performance was measured using a 13‐item measure adapted from the Individual Workplace Performance Questionnaire (IWPQ; task and contextual scales) [46]. Participants responded on 5‐point Likert scales (1 = *never* to 5 = *always*) with higher scores reflecting greater perceived work performance, which is an employee's perceived ability to engage in actions or behaviors that align with the goals of the organization [46].

##### Job satisfaction

Job satisfaction was measured using the 3‐item Job Satisfaction Scale from the Michigan Organizational Assessment Questionnaire [47]. Participants responded on 7‐point Likert scales (1 = *very strongly disagree* to 7 = *very strongly agree*) with higher scores reflecting greater job satisfaction, which is the extent to which an employee feels a sense of satisfaction and enjoyment in their job [48].

##### Perceived physical health

Perceived physical health was measured using a single‐item self‐rating of health measure, which reflects a person's perception of their current, general physical health status. Participants responded on a 5‐point Likert scale (1 = *poor* to 5 = *excellent*) with higher scores indicating better perceived physical health.

##### Turnover intentions

Turnover intentions were measured using a 3‐item scale developed by Tett and Meyer [49]. Participants responded on 7‐point Likert scales (1 = *very strongly disagree* to 7 = *very strongly agree*) with higher scores indicating greater intentions to leave the organization.

#### 2.3.5 Open‐ended questions

Three open‐ended questions were used to examine coping strategies used by participants. First, a broad question was asked, “What do you do to manage workplace demands and their impacts on your stress and well‐being?” This was followed by questions about specific practices including seeking professional support and taking sick leave, “How do you feel about the idea of seeking professional support for stress‐related problems if needed and are there any positive or negative consequences for seeking help?” and “What are your thoughts about taking sick leave when you are unwell or highly stressed?”. A fourth open‐ended question was used to examine participants' experiences of support being provided by the police organization, “What resources and support does your employer provide to help you to manage these demands and their impacts on your stress and well‐being?”

### 2.4 Data analysis

Quantitative survey data were initially analyzed using IBM SPSS Version 28. Missing data were estimated using expectation‐maximization (E‐M) imputation. Using Pearson's bivariate correlations, demographic characteristics, demands, and resources associated with stress‐related outcomes were examined to provide the strength and direction of linear relationships between variables. Partial least squares regression analyses were subsequently conducted using the *SEMinR* package [50] in *R* [51]. Partial least squares regression analyses were used because they are free from assumptions regarding the distribution of the data and are suitable for analyses with small sample sizes. Three regression analyses were conducted to examine predictors of each stress‐related outcome. The first regression analysis for each outcome included general demands as predictors, which were significantly associated (based on bivariate correlations) with the relevant outcome. The second regression analysis for each outcome included forensic‐specific demands as predictors, which were significantly associated with the relevant outcome. The third regression analysis for each outcome included resources as predictors, which were significantly associated with the relevant outcome. Two demographic characteristics, namely years of service in forensic services and gender, were controlled for in each regression model due to their significant association with many of the outcomes. Qualitative survey data were analyzed using NVivo 12. The qualitative data were examined using thematic analysis [52].

## 3 RESULTS

### 3.1 Associations between background characteristics and stress‐related outcomes

Bivariate correlation analyses were conducted testing the relationships between individual stress‐related outcomes and demographic characteristics. The demographic characteristics studied included role (i.e., police compared to staff members), rank, years of service, years of service in forensic services, centrally hosted compared to attached to a region, and gender (see Table S3). The analyses revealed that only two variables, namely years of service in forensic services and gender, produced significant correlations with individual stress‐related outcomes. The findings indicate that as the years of service in forensic services increase (small/medium positive correlations), officers' reports of burnout, psychological distress, and vicarious trauma also tend to increase. For gender (small/medium correlations), female officers reported lower levels of physical health compared to male officers.

### 3.2 Associations between general demands and stress‐related outcomes

#### 3.2.1 Bivariate correlations

The findings revealed that stress related to organizational and operational stress produced the strongest correlations with all measured outcomes. Specifically, greater stress in these domains was associated with lower work engagement, work performance, job satisfaction, and perceived physical health. In the same way, greater stress was related to increased psychological distress, vicarious trauma, burnout, and turnover intentions.

The results revealed that stress due to traumatic experiences had significant correlations with all outcomes except for turnover intentions, work performance, and job satisfaction. This indicates that experiencing greater trauma stress at work was associated with increased psychological distress, vicarious trauma, and burnout, as well as decreased work engagement and perceived physical health. Bivariate correlations are presented in Table S4, and regression results outlined below are presented in Tables S5 and S6.

#### 3.2.2 Predictors of burnout (exhaustion)

The results indicated one significant predictor of burnout, with the overall model explaining 56% of the variance in burnout scores. That is, operational stress (*β* = 0.64; 95% CI [0.43, 0.86]) made the strongest unique contribution to burnout, with higher levels of operational stress predicting greater burnout.

#### 3.2.3 Predictors of psychological distress

The results revealed that the general demands included in the regression model explained 42% of the variance in psychological distress. Operational stress (*β* = 0.40; 95% CI [0.14, 0.67]) emerged as the only significant contributor to this outcome, with higher levels of operational stress predicting greater psychological distress.

#### 3.2.4 Predictors of vicarious trauma

When assessing predictors of vicarious trauma, general demands explained 42% of the variance in this outcome. Organizational stress (*β* = 0.34; 95% CI [0.08, 0.57]) and operational stress (*β* = 0.34; 95% CI [0.10, 0.58]) independently emerged as significant predictors of vicarious trauma, with higher levels of organizational and operational stress predicting greater vicarious trauma.

#### 3.2.5 Predictors of turnover intentions

When assessing predictors of turnover intentions, the overall model explained 36% of the variance in this outcome. The only significant unique contributor to turnover intentions was organizational stress (*β* = 0.46; 95% CI [0.18, 0.73]), with greater organizational stress predicting increased turnover intentions.

#### 3.2.6 Predictors of work engagement

The results revealed that the general demands included in the regression model explained 37% of the variance in work engagement. Operational stress (*β* = −0.40; 95% CI [−0.74, −0.07]) emerged as the only significant predictor of work engagement, with lower operational stress predicting greater work engagement.

#### 3.2.7 Predictors of work performance

The current analysis identified no general demands as significant unique predictors of work performance.

#### 3.2.8 Predictors of job satisfaction

The general demands included in the regression model explained 45% of the variance in job satisfaction. Only one demand emerged as a significant contributor to job satisfaction. That is, increased organizational stress (*β* = −0.53; 95% CI [−0.73, −0.31]) predicted decreased job satisfaction.

#### 3.2.9 Predictors of perceived physical health

The current analysis identified no general demands as significant unique predictors of perceived physical health.

### 3.3 Associations between forensic‐specific demands and stress‐related outcomes

#### 3.3.1 Bivariate correlations

The majority of forensic‐specific demands had bivariate correlations with all or some of the stress‐related outcomes. The demands which were not associated with any stress‐related outcomes included being confronted with human suffering and working in dirty and physically demanding circumstances. Bivariate correlations are presented in Table S7, and regression results outlined below are presented in Tables S8–S15.

#### 3.3.2 Predictors of psychological distress

The results revealed that the forensic‐specific demands included in the regression model explained 39% of the variance in psychological distress. Two demands emerged as unique contributors to psychological distress. Specifically, being overwhelmed due to administrative obligations (*β* = 0.24; 95% CI [0.03, 0.44]) and concerns that colleagues' skills or drive negatively impact their work standards (*β* = 0.30; 95% CI [0.09, 0.51]) made the strongest unique contributions to the experience of psychological distress.

#### 3.3.3 Predictors of vicarious trauma

Similarly, results revealed that the forensic‐specific demands included in the regression model explained 46% of the variance in vicarious trauma. Among the demands, three predictor variables uniquely contributed to the experience of vicarious trauma. These demands were being overwhelmed due to administrative obligations (*β* = 0.28; 95% CI [0.09, 0.46]), the notion of letting down the team due to one's pace (*β* = 0.28; 95% CI [0.12, 0.46]), and years of service in forensic services (*β* = 0.19; 95% CI [0.04, 0.34]).

#### 3.3.4 Predictors of burnout (exhaustion)

The regression model used to predict burnout indicated that 48% of the variance in this outcome was explained by forensic‐specific demands. The two demands that most strongly predicted burnout were the hours of work that are perceived to impact work–life balance (*β* = 0.17; 95% CI [0.01, 0.31]) and years of service in forensic services (*β* = 0.21; 95% CI [0.03, 0.38]).

#### 3.3.5 Predictors of turnover intentions

No significant unique predictors of turnover intentions were identified in the analysis.

#### 3.3.6 Predictors of work engagement

The regression model used to predict work engagement indicated that 35% of the variance in this outcome was explained by forensic‐specific demands. Moreover, the results suggest that being overwhelmed due to administrative obligations (*β* = −0.25; 95% CI [−0.46, −0.02]), doubts about the thoroughness of one's investigation (*β* = −0.31; 95% CI [−0.59, −0.01]), and the notion of letting the team down due to one's pace (*β* = −0.18; 95% CI [−0.35, −0.01]) were the strongest predictors of work engagement. That is, lower perceptions of these demands were associated with increased work engagement.

#### 3.3.7 Predictors of work performance

The model used to predict work performance indicated that 27% of the variance in this outcome was explained by forensic‐specific demands. The only significant unique contributor to work performance was the notion of letting the team down due to one's pace (*β* = −0.23; 95% CI [−0.38, −0.06]). In other words, as the feeling of letting down the team decreased, work performance increased.

#### 3.3.8 Predictors of job satisfaction

When assessing predictors of job satisfaction, forensic‐specific demands explained 38% of variance in this outcome. Job satisfaction was predicted most strongly by overwhelm due to administrative obligations (*β* = −0.24; 95% CI [−0.42, −0.05]) and doubts about the thoroughness of one's investigation (*β* = −0.36; 95% CI [−0.63, −0.04]). That is, lower scores in these demands predicted greater job satisfaction.

#### 3.3.9 Predictors of perceived physical health

No significant unique predictors of perceived physical health were identified in the analysis.

### 3.4 Associations between resources and stress‐related outcomes

#### 3.4.1 Bivariate correlations

The presence of a psychosocial safety climate at the organizational level as well as at the overviewing officer level was moderately to strongly correlated with all outcome variables. That is, experiencing this climate at work was related to greater work engagement, work performance, job satisfaction, and perceived physical health. Moreover, this resource was associated with decreased psychological distress, vicarious trauma, burnout, and turnover intentions. This indicates that psychosocial safety climate may be a key protective factor.

Perceived direct supervisor support also produced independent, moderate to strong correlations with all outcome variables with the exception of perceived physical health. These findings suggest that receiving support from direct supervisors is linked with decreased psychological distress, vicarious trauma, burnout, and turnover intentions, as well as increased work engagement, work performance, and job satisfaction.

Perceived peer support was also correlated with all outcome variables except for work performance. This suggested that greater support from peers was associated with increased work engagement, job satisfaction, and perceived physical health, and decreased psychological distress, vicarious trauma, burnout, and turnover intentions. Perceived family support produced correlations with all outcome variables except for work performance and turnover intentions. Hence, similar to peer support, family support was associated with increased work engagement, job satisfaction, and perceived physical health, and decreased psychological distress, vicarious trauma, and burnout. Bivariate correlations are presented in Table S4, and regression results outlined below are presented in Tables S16 and S17.

#### 3.4.2 Predictors of burnout (exhaustion)

The resources included in the regression model explained 35% of the variance in burnout. Higher levels of supervisor support (*β* = −0.20; 95% CI [−0.40, −0.01]) and peer support (*β* = −0.28; 95% CI [−0.47, −0.08]) were identified as significant independent predictors of decreased burnout.

#### 3.4.3 Predictors of psychological distress

The resources included in the regression model explained 24% of the variance in psychological distress. Supervisor support (*β* = −0.24; 95% CI [0.43, 0.03]) was identified as the only unique contributor to this outcome, with higher perceived support from supervisors predicting lower psychological distress.

#### 3.4.4 Predictors of vicarious trauma

The resources included in the regression model explained 29% of the variance in vicarious trauma. Supervisor support (*β* = −0.33; 95% CI [−0.50, −0.13]) emerged as the only significant contributor to vicarious trauma, with higher levels of supervisor support predicting lower vicarious trauma.

#### 3.4.5 Predictors of turnover intentions

The resources included in the regression model explained 26% of the variance in turnover intentions. Among potential resources, psychosocial safety climate at the overviewing officer level (*β* = −0.28; 95% CI [−0.52, −0.01]) was highlighted as the only significant contributor to turnover intentions. That is, greater perceived psychosocial safety climate predicted decreased turnover intentions.

#### 3.4.6 Predictors of work engagement

The resources included in the regression model explained 29% of the variance in work engagement. Supervisor support (*β* = 0.26; 95% CI [0.02, 0.48]) uniquely contributed to this outcome, with greater support predicting increased work engagement scores.

#### 3.4.7 Predictors of work performance

The resources included in the regression model explained 17% of the variance in work performance. Psychosocial safety climate at the organizational level (*β* = 0.23; 95% CI [0.01, 0.44]) was identified as a significant predictor of work performance, with greater psychosocial safety climate predicting increased work performance.

#### 3.4.8 Predictors of job satisfaction

The resources included in the regression model explained 40% of the variance in job satisfaction. Increased job satisfaction was significantly predicted by two separate resources, namely greater psychosocial safety climate at the organizational level (*β* = 0.30; 95% CI [−0.12, −0.45]) and supervisor support (*β* = 0.24; 95% CI [0.02, 0.44]).

#### 3.4.9 Predictors of perceived physical health

The resources included in the regression model explained 17% of the variance in perceived physical health. The only significant unique contributor to perceived physical health was peer support (*β* = 0.24; 95% CI [0.04, 0.41]), with greater peer support predicting better perceived physical health.

### 3.5 Qualitative analysis

#### 3.5.1 Coping strategies used by forensic staff to manage demands

The survey included a number of open‐ended questions about coping strategies used by forensic staff. A qualitative analysis of these responses indicated that forensic staff use a diverse range of strategies to manage their job demands and associated stress. The coping strategies employed were divided into four categories. All percentages are based on the proportion of respondents who answered the question (56 out of 114 respondents answered this question).

##### Category 1: Psychological recovery and prioritizing physical health

Forensic staff reported using a range of coping strategies that are likely to promote psychological recovery and/or improve physical health. The most reported strategy was engaging in exercise (36%). This was followed by engaging in activities and hobbies outside of work (25%), such as travel, spending time away with family, dinners with friends, and hobbies such as gardening. A smaller number of personnel reported prioritization of eating a healthy diet (9%), getting enough sleep (5%), and efforts to broadly take care of their health (4%).

##### Category 2: Problem‐focused organization at work

Forensic staff reported using a range of problem‐focused strategies that can optimize or reduce the pressure associated with their time at work. The most commonly reported strategy was organizing time and prioritizing urgent work (16%). This was followed by the strategy of focusing on one job at a time (5%). Some staff also reported increasing their hours of work to get through the required tasks (4%).

##### Category 3: Emotion‐focused cognitive and behavioral strategies

Forensic staff reported using a range of emotion‐focused strategies to manage their demands and associated stress. The most commonly reported strategies were the use of relaxation exercises or yoga (13%) and attempts to compartmentalize and separate work from home (13%). This was followed by trying to ignore the demands (5%), consuming alcohol (4%), or using humor (4%).

##### Category 4: Accessing social support

Forensic staff reported accessing several forms of social support to manage their demands and associated stress (15%). This included support from family and friends (8%), support from peers at work (4%), supervisor support (1%), and support from peer support officers (PSOs; 1%).

#### 3.5.2 Perceptions of support provided by the organization

Forensic staff were asked about what resources and support the organization provides to help them manage job demands and the impact of job demands on stress and well‐being. All percentages are based on the proportion of respondents who answered the question (59 out of 114 respondents answered this question).

Of the group, 17% of those who responded to the question indicated that the organization provides no support. The staff who indicated that support was available from the organization listed a range of resources. This included flexible work arrangements (15%), PSOs (15%), human service officers (HSOs; e.g., psychologists and social workers within the organization; 12%), supervisor support (12%), gym access (7%), online information and mental health screening (7%), and an early intervention treatment program (EITP; 5%). However, it should be noted that 67% of those who listed PSOs as a source of support raised issues around lack of trust and perceptions that they will not maintain confidentiality. Additionally, all personnel who listed HSOs as a support service raised issues with the system. The issues included lack of access or not feeling comfortable with the person in the role. Other supports were also described, including weekly “e hour” (5%; an hour each week where exercise can be done during work time if workload permits), the organization providing information about services (3%), general training (3%), psychologists (3%), and well‐being sessions/conversations (3%).

#### 3.5.3 Attitudes of forensic staff toward help‐seeking

Forensic staff were asked how they felt about the idea of seeking professional support for stress‐related problems if needed and whether there were any positive or negative consequences for seeking help. All percentages are based on the proportion of respondents who answered the question (66 out of 114 respondents answered this question).

Most forensic staff who responded to the question reported that they were willing to see professional support if needed (59%). However, 30% reported that there was stigma associated with doing so. This included negative comments from colleagues or management, concerns around confidentiality, and perceptions that it would negatively affect their career. Of those responding to this question, 11% reported other barriers to accessing support such as lack of suitable in‐house services, cost, and the requirement to seek help in personal time. Of the group, 9% reported that they had no concern about consequences, while 5% reported that support was not needed. A small percentage indicated that they would not seek professional support (3%). Of the respondents to this question, 3% of staff reported that they had previously sought professional support, but that it did not work.

#### 3.5.4 Attitudes of forensic staff toward sick leave, including managing stress and well‐being

Forensic staff were asked about their thoughts regarding access to sick leave when they are unwell or highly stressed. All percentages are based on the proportion of respondents who answered the question (70 out of 114 respondents answered this question).

Most forensic staff, who responded to the question, reported that sick leave should be taken if it is needed (60%). Of this group, 37% did not specify whether they were referring to sick leave for the purposes of a physical illness or stress. For those that specified reasons, 14% explicitly said that they support using sick leave for stress, and 9% explicitly said that sick leave should only be used when unwell and not for stress. Despite quite a positive endorsement of the use of sick leave, 13% of respondents indicated that they felt they would be letting the team down if they took sick leave or that they could not take sick leave due to workload. A further 9% reported that they would not take sick leave. Of the respondents to this question, 7% indicated that there should be a special leave to accommodate work‐related stress. In respect to stigma, 7% reported that there was stigma associated with taking sick leave. A small percentage of the group indicated that sick leave is difficult to access due to the requirement for supporting documentation (3%) and that sick leave is the only option [when unwell or highly stressed] (3%).

## 4 DISCUSSION

The current study aimed to examine the demands and resources associated with stress‐related outcomes among forensic staff. We found that general job demands, forensic specific job demands, and resources were able to explain large portions of variance in work engagement, job satisfaction, psychological distress, vicarious trauma, burnout, and turnover intentions. Forensic‐specific demands also explained a large portion of variance in perceived work performance. In addition, general demands, forensic specific demands, and resources explained a medium portion of variance in perceived work performance and perceived physical health. The study also aimed to examine forensic staff descriptions of their coping strategies, their perceptions of organizational support resources, and attitudes toward help‐seeking and using sick leave for managing well‐being.

### 4.1 Job demands

In terms of general demands, trauma stress did not have any predictive relationships with any of the outcomes studied. Rather, all negative stress‐related outcomes are predicted by either organizational or operational stressors. Operational stress appears to be critically important when considering health outcomes, specifically psychological distress and burnout. If we are to tackle vicarious trauma, it is likely that both organizational and operational stressors need to be addressed. In terms of work‐related outcomes, organizational stressors were the only general job demand category that predicted job satisfaction and intention to turnover, and only operational stress predicted work engagement. Considering forensic specific demands, there were several key predictors of various positive and negative stress‐related outcomes. These included overwhelming administrative obligations, feeling that one will let the team down due to work pace, doubting the thoroughness of investigations, hours of work impacting balancing work–life balance, and concern about colleagues' skills or drive impacting their work standard.

While the traumatic nature of police work is likely to have some impact, its impact is overshadowed by both general and forensic‐specific demands that are related to operational and organizational stressors. This is consistent with prior research examining the proportion of harm associated with various sources of stress among police more broadly. Drew and Williamson [8] found that compared to trauma stress, burnout, and psychological distress were predicted three times more strongly by organizational stress, and two times more strongly by operational stress. Based on the current data, even in the specialist function of forensic services where trauma prevalence is likely to be higher compared to other parts of the police agency, we conclude that the most important levers in increasing well‐being and positively impacting the experience of work may not be the continued emphasis on interventions and strategies that are trauma‐focused, but rather to take a more holistic approach to supporting all types of demands.

Returning to the specific forensic demands, the demand that predicted most outcomes was overwhelming administrative obligations. In the current study, this demand emerged as a strong and unique predictor of increased psychological distress, higher levels of vicarious trauma, lower work engagement, and decreased job satisfaction. With the exception of intention to turnover, this demand was at a minimum correlated with all other outcomes. These findings are consistent with the limited body of previous research undertaken in forensic work contexts that has identified administrative accumulation as a key work‐related stressor [32].

The stress experienced by forensic staff resulting from the administrative demands of their jobs is likely to be associated with the stress forensic staff reported from feeling that they might let down the team due to one's work pace. This feeling is a key predictor of higher levels of vicarious trauma, less work engagement, and decreased work performance. It was further correlated with higher psychological distress, increased burnout, and low levels of job satisfaction.

Further exploring the theme of what type of stressors cause lower psychological health, work attitudes and performance in forensic staff, a unique predictive relationship was found between “concern about colleagues” skills or drive negatively impacting on one's own work standard and psychological distress. Except for work performance, this specific job demand was, at a minimum associated with all other outcomes that were studied. Forensic staff were also concerned about their own performance. The demand of “doubting the thoroughness of one's investigation” uniquely predicted decreased work engagement and reduced job satisfaction and was correlated with all negative stress‐outcomes, that is, psychological distress, vicarious trauma, burnout, and intention to turnover. Other specific demands, such as “worrying about missing something in examination”; “rushing examinations due to time pressure”; “worrying about amount of jobs team has each day” while not demonstrating predictive relationships with the outcomes studied, had significant bivariate correlations with a large number of the outcomes.

Across the findings related to forensic specific demands, the job demands predominately relate to time pressure and workload. It is possible that the ability to conduct thorough investigations may be influenced by other job aspects such as workload and time pressure. Therefore, providing officers with resources (e.g., time, manageable workload) to conduct thorough and high‐quality investigations may be beneficial in improving engagement and satisfaction in their work. Further, forensic staff seem to be impacted by both the demands they are experiencing personally and the demands that relate to the interaction between themselves and their colleagues. The results also highlight the need to consider the impact of work beyond the workplace. It is of note that the demand of “hours of work impacting on work–life balance” uniquely predicted burnout and was correlated with psychological distress, vicarious trauma, turnover intentions, work engagement and job satisfaction. This is consistent with prior research which has found shift work to be associated with increased occupational stress in police [53]. This highlights the need to consider strategies for mitigating the impact of long and variable work hours on well‐being among forensic staff.

### 4.2 Resources

The findings related to job demands, whether they be general or forensic specific, have identified the key stressors within the forensic context that are associated with lower psychological health, work attitudes, and performance in forensic staff. Guided by the JD‐R model of occupational stress, reducing these demands is essential in having a positive impact on officers' personal and work‐related well‐being, productivity, and longevity in the profession. This research finds supervisor support is likely to be the most impactful resource in counterbalancing or offsetting the job demands experienced by forensic staff. It has a unique predictive effect on five of the eight stress‐related outcomes (psychological distress, vicarious trauma, burnout, work engagement, and job satisfaction). This finding demonstrates the key role supervisors can play in mitigating harms and improving workplace outcomes for their staff. The importance of leader support was also found at the overviewing officer and the organization level, specifically for reducing psychosocial demands and risks in the workplace (measured via psychosocial safety climate). Psychosocial safety climate at the organizational level uniquely predicted work performance and job satisfaction, and psychosocial safety climate at the overviewing officer level uniquely predicted turnover intentions. When examining these findings, it is interesting to note that direct supervisor support was related to a mix of psychological outcomes and work attitude outcomes, while PSC was only related to work attitudes and performance. This suggests that in addition to increasing direct supervisor support, it may be important to seek to increase perceptions of psychosocial safety climate through the implementation of initiatives that demonstrate a commitment to staff well‐being. Emerging research in the manufacturing industry has demonstrated that training managers in psychosocial risk assessment across multiple levels of management can increase employee reports of psychosocial safety climate at the organization level [54]. Recently, programs to identify and address psychosocial hazards and risks have been developed for police agencies. These programs seek to uplift capacity across employee and management levels in identifying and mitigating the impact of psychosocial hazards and risks that are most relevant in police organizations. In turn, this is likely to positively impact the perceptions held by police personnel in regard to their psychosocial safety [55].

Similar to prior studies with police and other first responders [56, 57], peer support is an important resource for forensic staff in this cohort. However, peer support was only uniquely predictive of both burnout and perceived physical health, a much more limited range of outcomes compared to supervisor and leader supports. Regardless, this highlights that there is some value in encouraging peers to support each other both informally and through formal processes such as designated peer support officer roles.

### 4.3 Coping strategies

As discussed, a key aim of the current study was to identify key demands and resources that may inform efforts to improve health and well‐being among forensic staff. It also sought to examine forensic staff descriptions of their coping strategies, their perceptions of organizational support resources, and attitudes toward help‐seeking and using sick leave for managing well‐being.

In respect to help‐seeking, the findings collated from the qualitative data identified that only 59% of staff were willing to seek professional support for well‐being if needed. Rates of help‐seeking is similar to recent research in other police organizations [26, 58]. Various options for support (e.g., supervisor support, EITP, flexible work arrangements, were each reported by up to one fifth of staff), while 17% explicitly reported that no support was provided. However, as expected, and as an explanation for the low rates of help‐seeking, about one third of forensic staff reported help‐seeking stigma [58]. While willingness to seek help among police is growing [59], the current research highlights that investment in strategies to encourage help‐seeking among forensic staff and identify possible solutions to overcoming barriers and concerns associated with support is a priority. Less than one fifth of staff reported that it was appropriate to use sick leave to manage stress. Barriers to taking sick leave included stigma, letting the team down, and perceptions about whether it is appropriate. Forensic staff managers should consider their approach to encouraging staff to take a break from demands and stressors. While sick leave is an option and in some cases might be encouraged, other mechanisms for work breaks are preferable and should be considered. This may be the opportunity to move into a less stressful job role in the forensics area or a rotation out for specified periods of time. Such approaches have been suggested for other high‐risk groups in policing such as child abuse investigators [60, 61].

A range of coping strategies were reported by forensic staff in managing their work‐related stress and demands. As reported, strategies were classified into four broad categories including, psychological recovery and prioritizing physical health; use of problem‐focused coping strategies; use of emotion‐focused coping strategies; and, coping through seeking support. The themes identified reflect those of existing research in forensic contexts [10, 30, 32]. Of the staff who answered this question, psychological recovery and prioritizing physical health strategies were endorsed by largest number of staff, in particular: engaging in exercise (36%) and activities and hobbies outside of work (25%), such as travel, spending time away with family, dinners with friends, and hobbies such as gardening. Smaller numbers of staff reported using problem‐focused strategies (16% actively organized time and prioritized urgent work), emotion‐focused strategies (13% reported relaxation or yoga and 13% reported compartmentalizing work from home), and accessing social support (15% used some form of social support). While the coping strategies reported generally aligned to common categorisations of strategies (e.g., problem‐focused coping; emotion‐focused coping; seeking support; psychological recovery), they were described with contextual relevance to forensic job roles (e.g., active organization of time and prioritizing urgent work). This information may support the development of forensic personnel specific resources with direct relevance to these staff to support the uptake of healthy and effective coping strategy use.

### 4.4 Strengths, limitations, and future directions

The current study has several strengths that enhance our understanding of how to prevent negative stress‐related outcomes and promote positive stress‐related outcomes among forensic staff. Notably, the research is theory‐based through being grounded in the job demands–resources model and examined a broad range of demands, resources, and positive and negative outcomes. The sample was evenly gender balanced, and forensic staff were sampled across ranks and job roles. This is also one of the only Australian studies of forensic personnel. However, some limitations of the study should be noted. While the response rate of approximately 19% is an adequate sample size to draw conclusions with some confidence, a larger sample would improve the veracity with which conclusions and recommendations could be made. Additionally, while forensic staff within the sampled organization include both police and civilian staff members, the sample obtained was predominately sworn police personnel. Prior research has indicated that civilian staff members in police organizations may feel that they hold lower status in the organization by virtue of not being a sworn police officer [62]. While we did not find an association between sworn status and any of the outcomes, a larger sample would allow comparisons between these groups and observation of any differences that may exist. While we were able to draw conclusions about predictive relationships between demands and outcomes, the current study did adopt a cross‐sectional design. More robust conclusions regarding cause and effect could be achieved by a longitudinal research project. Future intervention research should also seek to focus on optimization of demands and uplifting of resources such as the capacity of supervisors to provide support and should evaluate the impact of these approaches.

## 5 CONCLUSION

The current research aimed to examine the factors associated with stress‐related outcomes among forensic staff. Results revealed a key role of occupational and organizational stressors in predicting a range of stress‐related outcomes. Turning to forensic‐specific demands, key predictors of stress‐related outcomes included overwhelming administrative obligations, feeling that one will let the team down due to work pace, doubting the thoroughness of investigations, hours of work impacting balancing work–life balance, and concern about colleagues' skills or drive impacting their work standard. While personnel working in forensic roles have traditionally been considered high risk due to their exposure to traumatic situations, it is important to highlight that organizational and operational stress, and not trauma stress, predicted the largest number of stress‐related outcomes. In terms of resources that can help to buffer the impact of demands, the results highlighted a key role of supervisor support in predicting stress‐related outcomes. Peer support and psychosocial safety climate were also important in predicting various stress‐related outcomes. Further research is needed to test these relationships longitudinally and to examine the impact of intervening upon demands and resources to improve both positive and negative stress‐related outcomes, such as well‐being, engagement, and performance in forensic staff.

## FUNDING INFORMATION

The project initially received funding from the Forensic Services Group, Queensland Police Service through a grant provided to Dr. Jacob Keech. Contribution to the project (through the provision of state‐wide employee survey data) was funded as part of an Australian Research Council (ARC) Linkage (LP200200834) grant “An Early Warning System for Police Workplace Health and Performance.” Project investigators: Associate Professor Jacqueline Drew, Griffith Criminology Institute, Griffith University; Professor Janet Ransley, Griffith Criminology Institute, Griffith University; and Commissioner Katarina Carroll, Queensland Police Service.

## CONFLICT OF INTEREST STATEMENT

The authors have no conflicts of interest to report.

## ETHICAL APPROVAL

Ethical approval for the project was granted by the Griffith University Human Research Ethics Committee (reference no: 2021/793) and the University of the Sunshine Coast Human Research Ethics Committee (reference no: A221725). Ethical approval for this research follows the Australian Code for the Responsible Conduct of Research 2018 and complies with the Declaration of Helsinki. Approval to conduct of the research was obtained from the Queensland Police Service Research Committee (QPSRC).

## CONSENT TO PARTICIPATE

Consent to participate was provided by all participants prior to participation.

## CONSENT TO PUBLISH

Consent to publish was provided by the Queensland Police Service Research Committee (QPSRC).

## Supporting information


Data S1.

## Data Availability

The data that support the findings of this study are available on request from the corresponding author. The data are not publicly available due to privacy or ethical restrictions.

## References

[1] Randall C , Buys N . Managing occupational stress injury in police services: a literature review. Int Public Health J. 2013;5(4):413–425.

[2] Mustafa M , Illzam E , Muniandy R , Hashmi M , Sharifa A , Nang M . Causes and prevention of occupational stress. IOSR J Dent Med Sci. 2015;14(11):98–104. 10.9790/0853-1411898104

[3] Ganster DC , Rosen CC . Work stress and employee health: a multidisciplinary review. J Manag. 2013;39(5):1085–1122. 10.1177/0149206313475815

[4] Bakker AB , Demerouti E . The job demands‐resources model: state of the art. J Manag Psychol. 2007;22(3):309–328. 10.1108/02683940710733115

[5] Collins PA , Gibbs ACC . Stress in police officers: a study of the origins, prevalence and severity of stress‐related symptoms within a county police force. Occup Med. 2003;53(4):256–264. 10.1093/occmed/kqg061 12815123

[6] Drew JM , Keech JJ , Martin S . Will I stay or will I go? Exploring job demand stress, organizational justice, and psychological health in decisions to leave the police agency or profession. Am J Crim Justice. 2025. 10.1007/s12103-025-09833-8 Epub 2025 Jul 15.

[7] Drew JM , Chevroulet C . Broken promises in policing: understanding leadership, procedural justice and psychological health through the lens of psychological contract breach. Policing. 2025;48(4):792–812. 10.1108/PIJPSM-05-2024-0082

[8] Drew JM , Williamson H . Trauma, critical incidents, organizational and operational stressors: the relationship between harms and psychological outcomes for police. Police Q. 2025;28(3):287–312. 10.1177/10986111241275048

[9] Foley J , Massey KLD . The ‘cost’ of caring in policing: from burnout to PTSD in police officers in England and Wales. Police J. 2021;94(3):298–315. 10.1177/0032258X20917442

[10] Salinas CR , Webb HE . Occupational stress and coping mechanisms in crime scene personnel. Occup Med. 2018;68(4):239–245. 10.1093/occmed/kqy03011 29579281

[11] Tehrani N . The role of psychological surveillance in reducing harm and building resilience in police forensic investigators. Police J. 2024;97(1):191–202. 10.1177/0032258X231151996

[12] García‐Herrero S , Mariscal MA , Gutiérrez JM , Ritzel DO . Using Bayesian networks to analyze occupational stress caused by work demands: preventing stress through social support. Accid Anal Prev. 2013;57:114–123. 10.1016/j.aap.2013.04.009 23672926

[13] Lazarus RS , Launier R . Stress‐related transactions between person and environment. In: Pervin LA , Lewis M , editors. Perspectives in interactional psychology. Boston, MA: Springer; 1978. p. 287–327.

[14] Cocker F , Sanderson K , LaMontagne A . Estimating the economic benefits of eliminating job strain as a risk factor for depression. J Occup Environ Med. 2017;59(1):12–17. 10.1097/JOM.0000000000000908 28045792

[15] Wagner SL , White N , Fyfe T , Matthews LR , Randall C , Regehr C , et al. Systematic review of posttraumatic stress disorder in police officers following routine work‐related critical incident exposure. Am J Ind Med. 2020;63(7):600–615. 10.1002/ajim.23120 32419181

[16] Papazoglou K , Andersen JP . A guide to utilizing police training as a tool to promote resilience and improve health outcomes among police officers. Traumatology. 2014;20(2):103–111. 10.1037/h0099394

[17] Beyond Blue . Answering the call national survey – National mental health and wellbeing study of police and emergency services, final report. Melbourne, Australia: Beyond Blue; 2018.

[18] Drew JM , Martin S . Community relations, workplace stress and well‐being in the context of mass demonstrations, defunding, and anti‐police sentiment: a national study of the experiences of United States law enforcement. In: Ricciardelli R , MacDermid JC , Ferguson L , editors. Occupational stress injuries: operational and organizational stressors among public safety personnel. Oxford, UK: Routledge; 2025. p. 48–75.

[19] Maslach C , Schaufeli WB , Leiter MP . Job burnout. Annu Rev Psychol. 2001;52:397–422. 10.1146/annurev.psych.52.1.397 11148311

[20] Bakker AB , Albrecht S . Work engagement: current trends. Career Dev Int. 2018;23(1):4–11. 10.1108/13620431011066231

[21] Schaufeli WB . Applying the job demands‐resources model: a ‘how to’ guide to measuring and tackling work engagement and burnout. Organ Dyn. 2017;46(2):120–132. 10.1016/j.orgdyn.2017.04.008

[22] Dollard MF , Bakker AB . Psychosocial safety climate as a precursor to conducive work environments, psychological health problems, and employee engagement. J Occup Organ Psychol. 2010;83(3):579–599. 10.1348/096317909X470690

[23] Pavšič Mrevlje T , Erčulj VI . Coping strategies and physical health in police units dealing with serious crime: does work experience count? Policing. 2021;15(3):1832–1841. 10.1093/police/paab006

[24] Reavley NJ , Too LS , Milner AJ , LaMontagne AD , Martin A , Papas A , et al. Depression literacy and help‐seeking in Australian police. Aust N Z J Psychiatry. 2018;52(11):1063–1074. 10.1177/0004867417753550 29402134

[25] Kyron MJ , Rikkers W , Bartlett J , Renehan E , Hafekost K , Baigent M , et al. Mental health and wellbeing of Australian police and emergency services employees. Arch Environ Occup Health. 2022;77(4):282–292. 10.1080/19338244.2021.1893631 33653231

[26] Carleton RN , Affifi TO , Taillieu T , Turner S , Mason JE , Ricciardelli R , et al. Assessing the relative impact of diverse stressors among public safety personnel. Int J Environ Res Public Health. 2020;17(4):1234. 10.3390/ijerph17041234 32075062 PMC7068554

[27] Baka L . Types of job demands make a difference. Testing the job demand‐control‐support model among polish police officers. Int J Hum Resour Manag. 2020;31(18):2265–2288. 10.1080/09585192.2018.1443962

[28] McCreary DR , Thompson MM . Development of two reliable and valid measures of stressors in policing: the operational and organizational police stress questionnaires. Int J Stress Manag. 2006;13(4):494–518. 10.1037/1072-5245.14.3.248

[29] Yoo YS , Cho OH , Cha KS , Boo YJ . Factors influencing post‐traumatic stress in Korean forensic science investigators. Asian Nurs Res (Korean Soc Nurs Sci). 2013;7(3):136–141. 10.1016/j.anr.2013.07.002 25030251

[30] Kelty SF , Gordon H . No burnout at this coal‐face: managing occupational stress in forensic personnel and the implications for forensic and criminal justice agencies. Psychiatry Psychol Law. 2015;22(2):273–290. 10.1080/13218719.2014.941092

[31] McKay‐Davis S , Robinson T , Sebetan IM , Stein P . Civilian forensic technician and sworn police officer job‐related stress. J Forensic Sci. 2020;65(6):2065–2070. 10.1111/1556-4029.14543 32790184

[32] Sollie H , Kop N , Euwema MC . Mental resilience of crime scene investigators: how police officers perceive and cope with the impact of demanding work situations. Crim Justice Behav. 2017;44(12):1580–1603. 10.1177/0093854817716959

[33] Adderley R , Smith LL , Bond Obe JW , Smith M . Physiological measurement of crime scene investigator stress. Int J Police Sci Manag. 2012;14(2):166–176. 10.1350/ijps.2012.14.2.274

[34] Jarero I , Uribe S . The EMDR protocol for recent critical incidents: follow‐up report of an application in a human massacre situation. J EMDR Pract Res. 2012;6(2):50–61. 10.1891/1933-3196.6.2.50

[35] Rosansky JA , Cook J , Rosenberg H , Sprague JE . PTSD symptoms experienced and coping tactics used by crime ccene investigators in the United States. J Forensic Sci. 2019;64(5):1444–1450. 10.1111/1556-4029.14044 30893487

[36] Pavšič Mrevlje T . Coping with work‐related traumatic situations among crime scene technicians. Stress Health. 2016;32(4):374–382. 10.1002/smi.2631 25641805

[37] Weiss DS , Brunet A , Best SR , Metzler TJ , Liberman A , Pole N , et al. Frequency and severity approaches to indexing exposure to trauma: the critical incident history questionnaire for police officers. J Trauma Stress. 2010;23(6):734–743. 10.1002/jts.20576 21171134 PMC3974917

[38] Dollard MF . The PSC‐4; a short PSC tool. In: Dollard MF , Dormann C , Awang Idris M , editors. Psychosocial safety climate: a new work stress theory. Cham, Switzerland: Springer International Publishing; 2019. p. 385–409.

[39] Vig KD , Mason JE , Carleton RN , Asmundson GJG , Anderson GS , Groll D . Mental health and social support among public safety personnel. Occup Med. 2020;70(6):427–433. 10.1093/occmed/kqaa129 PMC7566747 32705138

[40] Graham L , Brown N , Plater M , Gracey S , Legate N , Weinstein N . National policing wellbeing survey 2019: Summary of evidence and insights. Durham, UK: Durham University; 2020.

[41] Demerouti E , Bakker AB . The Oldenburg burnout inventory: a good alternative to measure burnout (and engagement). In: Halbesleben JRB , editor. Handbook of stress and burnout in health care. New York, NY: Nova Science Publishers; 2008. p. 65–78.

[42] Kessler RC , Andrews G , Colpe LJ , Hiripi E , Mroczek DK , Normand SLT , et al. Short screening scales to monitor population prevalences and trends in non‐specific psychological distress. Psychol Med. 2002;32(6):959–976. 10.1017/S0033291702006074 12214795

[43] Viertiö S , Kiviruusu O , Piirtola M , Kaprio J , Korhonen T , Marttunen M , et al. Factors contributing to psychological distress in the working population, with a special reference to gender difference. BMC Public Health. 2021;21:611. 10.1186/s12889-021-10560-y 33781240 PMC8006634

[44] Benuto L , Singer J , Cummings C , Ahrendt A . The vicarious trauma scale: confirmatory factor analysis and psychometric properties with a sample of victim advocates. Health Soc Care Community. 2018;26(4):564–571. 10.1111/hsc.12554 29488272

[45] Molnar BE , Sprang G , Killian KD , Gottfried R , Emery V , Bride BE . Advancing science and practice for vicarious traumatization/secondary traumatic stress: a research agenda. Dent Traumatol. 2017;23(2):129–142. 10.1037/trm0000122

[46] Koopmans L , Bernaards C , Hildebrandt V , van Buuren S , van der Beek AJ , de Vet HCW . Development of an individual work performance questionnaire. Int J Product Perform Manag. 2012;62(1):6–28. 10.1108/17410401311285273

[47] Cammann C , Fichman M , Jenkins GD , Klesh J . Michigan organizational assessment questionnaire. In: Seashore SE , Lawler EE , Mirvis PH , Cammann C , editors. Assessing organizational change: a guide to methods, measures, and practices. New York, NY: Wiley; 1983. p. 71–138.

[48] Holt TJ , Blevins KR , Smith RW . Examining the impact of organizational and individual characteristics on forensic scientists' job stress and satisfaction. J Crime Justice. 2017;40(1):34–49. 10.1080/0735648X.2016.1216731

[49] Tett RP , Meyer JP . Job satisfaction, organizational commitment, turnover intention, and turnover: path analyses based on meta‐analytic findings. Pers Psychol. 1993;46(2):259–293. 10.1111/j.1744-6570.1993.tb00874.x

[50] Hair JF Jr , Hult GTM , Ringle CM , Sarstedt M , Danks NP , Ray S , et al. The SEMinR package. Partial least squares structural equation modeling (PLS‐SEM) using R: a workbook. Cham, Switzerland: Springer Nature; 2021. p. 49–74.

[51] R Core Team . R: a language and environment for statistical computing [computer program]. Vienna, Austria: R Foundation for Statistical Computing; 2022.

[52] Braun V , Clarke V . Successful qualitative research: a practical guide for beginners. London, UK: Sage; 2013.

[53] Ma CC , Andrew ME , Fekedulegn D , Gu JK , Hartley TA , Charles LE , et al. Shift work and occupational stress in police officers. Saf Health Work. 2015;6(1):25–29. 10.1016/j.shaw.2014.10.001 25830066 PMC4372186

[54] Berglund RT , Kombeiz O , Dollard M . Manager‐driven intervention for improved psychosocial safety climate and psychosocial work environment. Saf Sci. 2024;176:106552. 10.1016/j.ssci.2024.106552

[55] Drew JM , Keech JJ . EMPOWER workplace wellbeing workshops: a wellbeing intervention for police staff, leaders and peer support networks. Pol Sci. 2024;9(2):28–29.

[56] Donovan N . Peer support facilitates post‐traumatic growth in first responders: a literature review. Dent Traumatol. 2022;24(4):277–285. 10.1177/14604086221079441

[57] Milliard B . Utilization and impact of peer‐support programs on police officers' mental health. Front Psychol. 2020;11:1686. 10.3389/fpsyg.2020.01686 32765375 PMC7381167

[58] Drew JM , Martin S . A national study of police mental health in the USA: stigma, mental health and help‐seeking behaviors. J Police Crim Psychol. 2021;36(2):295–306. 10.1007/s11896-020-09424-9

[59] Lane J , Le M , Martin K , Bickle K , Campbell E , Ricciardelli R . Police attitudes toward seeking professional mental health treatment. J Police Crim Psychol. 2021;37(1):123–131. 10.1007/s11896-021-09467-6

[60] Tehrani N . Psychological well‐being and workability in child abuse investigators. Occup Med. 2018;68(3):165–170. 10.1093/occmed/kqy016 29546431

[61] Kleineidam NJ , Fischbach A . Feeling with the victim: empathy for suffering as a job stressor in internet child exploitation investigation. J Police Crim Psychol. 2024. 10.1007/s11896-024-09720-8 Epub 2024 Dec 11.

[62] Orosco C , Gaub JE . “I am doing my part, you are doing your part”: the sworn‐civilian divide in police dispatching. Policing. 2023;46(1):164–178. 10.1108/PIJPSM-07-2022-0090

