# Supplementary material for: Workplace demands, resources, and well‐being among police staff working in forensic services
Source: J Forensic Sci. 2025 Sep 8;71(1):139–54. doi: 10.1111/1556-4029.70179 (PMC12766658; doi:10.1111/1556-4029.70179)
Supplement: Supplementary file 1 — Data S1. [file JFO-71-139-s001.docx]

# Online Supplementary Materials

TABLE S1 Participant demographic characteristics (N = 114).

| **Demographic characteristic** | ***N*** | **%** |
| --- | --- | --- |
| **Age** (*M* = 46.01; *SD* = 6.92; Range = 25– 59) |  |  |
| **Gender** |  |  |
| Female | 54 | 47.4 |
| Male | 59 | 51.8 |
| Missing | 1 | .8 |
| **Police or staff** |  |  |
| Sworn officer | 98 | 86.0 |
| Civilian staff | 16 | 14.0 |
| **Rank** |  |  |
| Constable | 1 | .9 |
| Senior Constable | 55 | 48.2 |
| Sergeant | 34 | 29.8 |
| Senior Sergeant | 6 | 5.3 |
| Inspector | 1 | .9 |
| Higher sworn rank than Chief Superintendent | 1 | .9 |
| AO3 (or equivalent) | 7 | 6.1 |
| AO5 (or equivalent) | 3 | 2.6 |
| AO6 (or equivalent) | 2 | 1.8 |
| Missing | 4 | 3.5 |
| **Job in forensic services** |  |  |
| Coronial Support | 3 | 2.6 |
| DNA Management and Quality Management | 3 | 2.6 |
| Electronic Evidence Management | 6 | 5.3 |
| Fingerprint Bureau | 13 | 11.4 |
| Forensic Imaging | 5 | 4.4 |
| Scenes of Crime | 59 | 51.8 |
| Scientific Section | 9 | 7.9 |
| Forensic Management, Scene Coordination | 2 | 1.8 |
| Forensic Crash Investigations | 14 | 12.3 |
| **Years of service** |  |  |
| Less than 5 years | 3 | 2.6 |
| 5 to 10 years | 6 | 5.3 |
| 11 to 15 years | 27 | 23.7 |
| 16 to 20 years | 28 | 24.6 |
| 21+ years | 50 | 43.9 |
| **Years of service in forensic services group** |  |  |
| Less than 1 year | 2 | 1.8 |
| 1 to 3 years | 9 | 7.9 |
| 4 to 6 years | 13 | 11.4 |
| 7 to 9 years | 12 | 10.5 |
| 10 years or more | 78 | 68.4 |
| **Centrally hosted; attached to other police station** |  |  |
| Centrally hosted | 27 | 23.7 |
| Attached to other police station | 87 | 76.3 |
| **Geographic location** |  |  |
| Centrally Hosted Brisbane - Work in PHQ or Similar (Excludes Brisbane Region) | 27 | 23.7 |
| Brisbane Region - North Brisbane District | 5 | 4.4 |
| Brisbane Region - South Brisbane District | 12 | 10.5 |
| Brisbane Region – Regional Office | 2 | 1.8 |
| Central Region - Capricornia District | 1 | .9 |
| Central Region - Mackay District | 4 | 3.5 |
| Central Region - Regional Office | 1 | .9 |
| Far Northern Region - North Police District | 6 | 5.3 |
| Far Northern Region - Regional Office | 2 | 1.8 |
| North Coast Region - Sunshine Coast District | 6 | 5.3 |
| North Coast Region - Wide Bay Burnett | 3 | 2.6 |
| North Coast Region - Moreton District | 9 | 7.9 |
| Northern Region - Mt Isa District | 1 | .9 |
| Northern Region - Townsville District | 12 | 10.5 |
| Southern Region - Darling Downs District | 6 | 5.3 |
| Southern Region – Ipswich District | 3 | 2.6 |
| Southern Region - South West District | 1 | .9 |
| Southern Region - Regional Office | 2 | 1.8 |
| South Eastern Region - Gold Coast District | 8 | 7.0 |
| South Eastern Region - Logan District | 3 | 2.6 |
| **Geographic location** |  |  |
| Centrally Hosted Brisbane - Work in PHQ or Similar (excludes Brisbane Region) | 27 | 23.7 |
| Brisbane region (North Brisbane District, South Brisbane District, Regional Office) | 19 | 16.7 |
| Central Region (Capricornia District, Mackay District, Regional Office) | 6 | 5.3 |
| Far Northern Region (North Police District, Regional Office) | 8 | 7.1 |
| North Coast Region (Sunshine Coast District, Wide Bay Burnett, Moreton District) | 18 | 15.8 |
| Northern Region (Mt Isa District, Townsville District) | 13 | 11.4 |
| Southern Region (Darling Downs District, District, South West District, Regional Office) | 12 | 10.6 |
| South Eastern Region (Gold Coast District, Logan District) | 11 | 9.6 |
| **Marital status** |  |  |
| Married/Living with partner | 92 | 80.7 |
| Single, but previously married, currently separated, or divorced | 9 | 7.9 |
| Single, never married | 13 | 11.4 |
| **Partner works at QPS or another first responder agency** |  |  |
| Partner does not work for QPS or another first responder agency | 74 | 64.9 |
| Not in a relationship | 17 | 14.9 |
| Partner in a police officer in the QPS | 19 | 16.7 |
| Partner in a staff member in the QPS | 1 | .9 |
| Partner works in another first responder agency (e.g., Fire, Ambulance) | 3 | 2.6 |

TABLE S2 Alpha reliability coefficients for study variables.

|  | **α** |
| --- | --- |
| Organisational stress | .94 |
| Operational stress | .96 |
| Trauma stress | .89 |
| Psychosocial safety climate - organisation | .84 |
| Psychosocial safety climate - overviewing officer | .97 |
| Supervisor support | .94 |
| Peer support | .96 |
| Family support^ | .86 |
| Psychological distress | .94 |
| Vicarious trauma | .85 |
| Work engagement | .76 |
| Work performance | .89 |
| Burnout/exhaustion | .87 |
| Job satisfaction | .90 |
| Turnover intentions | .87 |

^Spearman Brown correlation used for 2 item family support scale.

TABLE S3 Bivariate correlations between demographic factors and stress-related outcomes.

|  | **PD** | **VT** | **WE** | **WP** | **BE** | **JS** | **PH** | **TI** |
| --- | --- | --- | --- | --- | --- | --- | --- | --- |
| Sworn officers; civilian staff | -.03 | -.01 | -.03 | -.08 | -.03 | -.04 | -.11 | -.04 |
| Rank (categorical including staff) | -.02 | -.05 | .06 | .08 | -.03 | .06 | .13 | -.00 |
| Years of service | .11 | .12 | -.07 | -.06 | .16 | .02 | .06 | .02 |
| Years of service in Forensic Services Group | .20* | .21* | -.15 | -.12 | .26** | -.16 | -.17 | .11 |
| Centrally hosted; attached to other police station | .03 | .04 | .02 | .17 | .07 | -.01 | -.13 | .00 |
| Gender | .10 | .12 | -.18 | -.16 | .13 | -.15 | -.19* | .06 |

*Note*. * *p* < 0.05, ** *p* < 0.01; PD = Psychological distress; VT = Vicarious trauma; WE = Work engagement; WP = Work performance; BE = Burnout/exhaustion; JS = Job satisfaction; PH = Perceived physical health; TI = Turnover intentions.

TABLE S4 Bivariate correlations between demands, resources, and stress-related outcomes.

|  | **1** | **2** | **3** | **4** | **5** | **6** | **7** | **8** | **9** | **10** | **11** | **12** | **13** | **14** | **15** |
| --- | --- | --- | --- | --- | --- | --- | --- | --- | --- | --- | --- | --- | --- | --- | --- |
| 1. Organisational stress | - |  |  |  |  |  |  |  |  |  |  |  |  |  |  |
| 1. Operational stress | .80^**^ | - |  |  |  |  |  |  |  |  |  |  |  |  |  |
| 1. Trauma stress | .49^**^ | .59^**^ | - |  |  |  |  |  |  |  |  |  |  |  |  |
| 1. PSC-Org | -.46^**^ | -.43^**^ | -.17 | - |  |  |  |  |  |  |  |  |  |  |  |
| 1. PSC-Overviewing officer | -.53^**^ | -.42^**^ | -.23^*^ | .56^**^ | - |  |  |  |  |  |  |  |  |  |  |
| 1. Supervisor support | -.54^**^ | -.37^**^ | -.20 | .28^**^ | .51^**^ | - |  |  |  |  |  |  |  |  |  |
| 1. Peer support | -.30^**^ | -.15 | -.03 | .21^*^ | .32^**^ | .31^**^ | - |  |  |  |  |  |  |  |  |
| 1. Family support | -.37^**^ | -.33^**^ | -.16 | .31^**^ | .39^**^ | .35^**^ | .47^**^ | - |  |  |  |  |  |  |  |
| 1. Psychological distress | .58^**^ | .63^**^ | .43^**^ | -.29^**^ | -.38^**^ | -.38^**^ | -.24^*^ | -.33^**^ | - |  |  |  |  |  |  |
| 1. Vicarious trauma | .61^**^ | .61^**^ | .33^**^ | -.33^**^ | -.34^**^ | -.43^**^ | -.26^**^ | -.35^**^ | .58^**^ | - |  |  |  |  |  |
| 1. Work engagement | -.56^**^ | -.57^**^ | -.27^**^ | .35^**^ | .42^**^ | .41^**^ | .31^**^ | .28^**^ | -.47^**^ | -.57^**^ | - |  |  |  |  |
| 1. Work performance | -.30^**^ | -.31^**^ | -.12 | .37^**^ | .33^**^ | .23^*^ | .11 | .18 | -.31^**^ | -.37^**^ | .47^**^ | - |  |  |  |
| 1. Burnout/exhaustion | .64^**^ | .74^**^ | .38^**^ | -.26^**^ | -.35^**^ | -.38^**^ | -.46^**^ | -.42^**^ | .61^**^ | .74^**^ | -.74^**^ | -.26^**^ | - |  |  |
| 1. Job satisfaction | -.66^**^ | -.59^**^ | -.16 | .49^**^ | .49^**^ | .44^**^ | .36^**^ | .35^**^ | -.54^**^ | -.62^**^ | .80^**^ | .45^**^ | -.62^**^ | - |  |
| 1. Perceived physical health | -.31^**^ | -.36^**^ | -.21^*^ | .27^**^ | .20^*^ | .14 | .29^**^ | .19^*^ | -.17 | -.31^**^ | .49^**^ | .13 | -.47^**^ | .39^**^ | - |
| 1. Turnover intentions | .57^**^ | .52^**^ | .19 | -.41^**^ | -.47^**^ | -.33^**^ | -.19^*^ | -.17 | .45^**^ | .45^**^ | -.41^**^ | -.21^*^ | .45^**^ | -.65^**^ | -.16 |

*Note*. * *p* < 0.05, ** *p* < 0.01, *** *p* < .001 (all are N = 114 except trauma stress correlations which are N = 98).

PSC-Org = Psychosocial safety climate (organisation); PSC-Overviewing officer = Psychosocial safety climate (overviewing officer)

TABLE S5 Regression results for demands predicting positive stress-related outcomes.

|  | **β** | **95% CI (LB)** | **95% CI (UB)** | ***f*^2^** |
| --- | --- | --- | --- | --- |
| **Work engagement (*R*^2^ = .37)** |  |  |  |  |
| Organisational stress | -.272 | -.574 | .019 | .043 |
| Operational stress | -.400 | -.736 | -.068 | .079 |
| Trauma stress | .107 | -.107 | .301 | .011 |
| Years of service in Forensic Services Group | -.019 | -.144 | .113 | .000 |
| Gender | -.106 | -.253 | .044 | .018 |
| **Work performance (*R*^2^ = .10)** |  |  |  |  |
| Organisational stress | -.129 | -.495 | .236 | .008 |
| Operational stress | -.206 | -.559 | .149 | .016 |
| **Job satisfaction (*R*^2^ = .45)** |  |  |  |  |
| Organisational stress | -.525 | -.729 | -.313 | .181 |
| Operational stress | -.158 | -.401 | .089 | .016 |
| Years of service in Forensic Services Group | -.023 | -.160 | .112 | .001 |
| Gender | -.053 | -.182 | .085 | .005 |
| **Perceived physical health (*R*^2^ = .16)** |  |  |  |  |
| Organisational stress | -.025 | -.318 | .244 | .000 |
| Operational stress | -.282 | -.602 | .046 | .029 |
| Trauma stress | -.037 | -.243 | .177 | .001 |
| Years of service in Forensic Services Group | -.091 | -.273 | .112 | .009 |
| Gender | -.146 | -.308 | .024 | .024 |

*Note.* CI = Confidence interval; LB = Lower boundary; UB = Upper boundary.

TABLE S6 Regression results for demands predicting negative stress-related outcomes.

|  | **β** | **95% CI (LB)** | **95% CI (UB)** | ***f*^2^** |
| --- | --- | --- | --- | --- |
| **Psychological distress (*R*^2^ = .42)** |  |  |  |  |
| Organisational stress | .205 | -.022 | .442 | .026 |
| Operational stress | .404 | .135 | .671 | .091 |
| Trauma stress | .073 | -.101 | .234 | .007 |
| Years of service in Forensic Services Group | .059 | -.066 | .181 | .006 |
| Gender | .012 | -.131 | .149 | .000 |
| **Vicarious trauma (*R*^2^ = .42)** |  |  |  |  |
| Organisational stress | .335 | .082 | .574 | .066 |
| Operational stress | .341 | .101 | .583 | .065 |
| Trauma stress | -.036 | -.211 | .157 | .002 |
| Years of service in Forensic Services Group | .071 | -.072 | .210 | .009 |
| Gender | .030 | -.120 | .164 | .002 |
| **Burnout/exhaustion (*R*^2^ = .56)** |  |  |  |  |
| Organisational stress | .140 | -.063 | .346 | .016 |
| Operational stress | .640 | .431 | .863 | .291 |
| Trauma stress | -.076 | -.222 | .073 | .009 |
| Years of service in Forensic Services Group | .099 | -.026 | .218 | .02 |
| Gender | .040 | -.086 | .176 | .003 |
| **Turnover intentions (*R*^2^ = .36)** |  |  |  |  |
| Organisational stress | .461 | .182 | .728 | .116 |
| Operational stress | .249 | -.042 | .548 | .032 |
| Trauma stress | -.170 | -.378 | .046 | .030 |
| Years of service in Forensic Services Group | -.009 | -.149 | .137 | .000 |
| Gender | -.020 | -.172 | .125 | .000 |

*Note.* CI = Confidence interval; LB = Lower boundary; UB = Upper boundary.

TABLE S7 Bivariate correlations between forensic specific demands and stress-related outcomes.

|  | **PD** | **VT** | **WE** | **WP** | **BE** | **JS** | **PH** | **TI** |
| --- | --- | --- | --- | --- | --- | --- | --- | --- |
| a. Admin obligations overwhelm | .42*** | .48*** | -.44*** | -.24** | .40*** | -.43*** | -.17 | .32*** |
| b. Hours of work impact work life balance | .30** | .33*** | -.31*** | -.17 | .40*** | -.27** | -.12 | .21* |
| c. Confronted with human suffering | .16 | .16 | -.08 | .02 | .16 | -.10 | -.17 | .26** |
| d. Dirty and physically demanding circumstances | .18 | .11 | -.07 | -.10 | .10 | -.18 | .00 | .23* |
| e. Doubting own thoroughness in investigation | .41*** | .44*** | -.38*** | -.42*** | .41*** | -.46*** | -.17 | .36*** |
| f. Wondering about missing something in examination | .40*** | .39*** | -.24* | -.38*** | .28** | -.34*** | -.12 | .40*** |
| g. Concerned colleagues’ skills or drive negatively impact their work standard | .46*** | .38*** | -.29** | -.11 | .40*** | -.37*** | -.24** | .31*** |
| h. Accepting that sometimes cannot solve crime | .17 | .20* | -.28** | -.19* | .24* | -.16 | -.17 | -.06 |
| i. Challenges due to cooperation between police units | .17 | .26** | -.27** | -.21* | .25** | -.26** | -.13 | -.01 |
| j. Rush examinations due to time pressure | .49*** | .43*** | -.42*** | -.25** | .48*** | -.50*** | -.22* | .40*** |
| k. Sufficient people in office to complete workload | -.17 | -.20* | .20* | .19* | -.20* | .24* | .15 | -.19* |
| l. Pace letting down the team | .25** | .39*** | -.30** | -.31*** | .39*** | -.26** | -.11 | .15 |
| m. Worry about amount of jobs team has each day | .30** | .44*** | -.37*** | -.25** | .47*** | -.31*** | -.25** | .26** |

*Note*. * *p* < 0.05, ** *p* < 0.01, *** *p* < .001; PD = Psychological distress; VT = Vicarious trauma; WE = Work engagement; WP = Work performance; BE = Burnout/exhaustion; JS = Job satisfaction; PH = Perceived physical health; TI = Turnover intentions.

TABLE S8 Regression results for forensic staff specific demands predicting psychological distress (R^2^ = .39).

|  | **β** | **95% CI (LB)** | **95% CI (UB)** | ***f*^2^** |
| --- | --- | --- | --- | --- |
| a. Admin obligations overwhelm | .244 | .028 | .443 | .056 |
| b. Hours of work impact work life balance | .040 | -.151 | .206 | .002 |
| e. Doubting own thoroughness in investigation | .065 | -.233 | .364 | .002 |
| f. Wondering about missing something in examination | .143 | -.201 | .455 | .010 |
| g. Concerned colleagues’ skills or drive negatively impact their work standard | .295 | .093 | .511 | .072 |
| j. Rush examinations due to time pressure | .062 | -.196 | .340 | .003 |
| l. Pace letting down the team | .156 | -.025 | .338 | .030 |
| m. Worry about amount of jobs team has each day | -.126 | -.314 | .088 | .014 |
| Years of service in Forensic Services Group | .122 | -.073 | .315 | .021 |
| Gender | .050 | -.088 | .194 | .003 |

*Note.* CI = Confidence interval; LB = Lower boundary; UB = Upper boundary.

TABLE S9 Regression results for forensic staff specific demands predicting vicarious trauma (R^2^ = .46).

|  | **β** | **95% CI (LB)** | **95% CI (UB)** | ***f*^2^** |
| --- | --- | --- | --- | --- |
| a. Admin obligations overwhelm | .277 | .091 | .459 | .082 |
| b. Hours of work impact work life balance | .056 | -.106 | .215 | .005 |
| e. Doubting own thoroughness in investigation | .180 | -.165 | .480 | .016 |
| f. Wondering about missing something in examination | .042 | -.231 | .307 | .001 |
| g. Concerned colleagues’ skills or drive negatively impact their work standard | .182 | -.008 | .367 | .032 |
| j. Rush examinations due to time pressure | -.088 | -.293 | .117 | .005 |
| l. Pace letting down the team | .284 | .122 | .457 | .115 |
| m. Worry about amount of jobs team has each day | .046 | -.152 | .220 | .002 |
| Years of service in Forensic Services Group | .192 | .036 | .341 | .055 |
| Gender | .070 | -.089 | .228 | .007 |

*Note.* CI = Confidence interval; LB = Lower boundary; UB = Upper boundary.

TABLE S10 Regression results for forensic staff specific demands predicting burnout/exhaustion (R^2^ = .48).

|  | **β** | **95% CI (LB)** | **95% CI (UB)** | ***f*^2^** |
| --- | --- | --- | --- | --- |
| a. Admin obligations overwhelm | .092 | -.080 | .271 | .009 |
| b. Hours of work impact work life balance | .166 | .011 | .314 | .044 |
| e. Doubting own thoroughness in investigation | .266 | -.032 | .583 | .038 |
| f. Wondering about missing something in examination | -.222 | -.538 | .117 | .030 |
| g. Concerned colleagues’ skills or drive negatively impact their work standard | .108 | -.062 | .277 | .013 |
| j. Rush examinations due to time pressure | .103 | -.133 | .325 | .009 |
| l. Pace letting down the team | .275 | .111 | .442 | .112 |
| m. Worry about amount of jobs team has each day | .146 | -.037 | .335 | .022 |
| Years of service in Forensic Services Group | .210 | .030 | .378 | .065 |
| Gender | .056 | -.089 | .204 | .004 |

*Note.* CI = Confidence interval; LB = Lower boundary; UB = Upper boundary.

TABLE S11 Regression results for forensic staff specific demands predicting turnover intentions (R^2^ = .24).

|  | **β** | **95% CI (LB)** | **95% CI (UB)** | ***f*^2^** |
| --- | --- | --- | --- | --- |
| a. Admin obligations overwhelm | .153 | -.082 | .394 | .015 |
| b. Hours of work impact work life balance | .006 | -.184 | .197 | .000 |
| e. Doubting own thoroughness in investigation | -.040 | -.393 | .277 | .000 |
| f. Wondering about missing something in examination | .285 | -.070 | .649 | .031 |
| g. Concerned colleagues’ skills or drive negatively impact their work standard | .116 | -.115 | .341 | .009 |
| j. Rush examinations due to time pressure | .109 | -.109 | .336 | .007 |
| l. Pace letting down the team | .024 | -.135 | .197 | .000 |
| m. Worry about amount of jobs team has each day | -.014 | -.248 | .215 | .000 |
| Years of service in Forensic Services Group | .091 | -.127 | .294 | .008 |
| Gender | .021 | -.137 | .188 | .000 |

*Note.* CI = Confidence interval; LB = Lower boundary; UB = Upper boundary.

TABLE S12 Regression results for forensic staff specific demands predicting work engagement (R^2^ = .35).

|  | **β** | **95% CI (LB)** | **95% CI (UB)** | ***f*^2^** |
| --- | --- | --- | --- | --- |
| a. Admin obligations overwhelm | -.247 | -.464 | -.015 | .057 |
| b. Hours of work impact work life balance | -.055 | -.253 | .146 | .005 |
| e. Doubting own thoroughness in investigation | -.314 | -.593 | -.010 | .042 |
| f. Wondering about missing something in examination | .264 | -.059 | .552 | .034 |
| g. Concerned colleagues’ skills or drive negatively impact their work standard | -.063 | -.291 | .172 | .004 |
| j. Rush examinations due to time pressure | -.087 | -.390 | .240 | .005 |
| l. Pace letting down the team | -.177 | -.345 | -.010 | .037 |
| m. Worry about amount of jobs team has each day | -.074 | -.323 | .170 | .003 |
| Years of service in Forensic Services Group | -.128 | -.258 | .005 | .021 |
| Gender | -.088 | -.232 | .064 | .011 |

*Note.* CI = Confidence interval; LB = Lower boundary; UB = Upper boundary.

TABLE S13 Regression results for forensic staff specific demands predicting work performance (R^2^ = .27).

|  | **β** | **95% CI (LB)** | **95% CI (UB)** | ***f*^2^** |
| --- | --- | --- | --- | --- |
| a. Admin obligations overwhelm | -.046 | -.268 | .196 | .002 |
| b. Hours of work impact work life balance | -.011 | -.208 | .182 | .000 |
| e. Doubting own thoroughness in investigation | -.282 | -.613 | .047 | .031 |
| f. Wondering about missing something in examination | -.166 | -.518 | .166 | .010 |
| g. Concerned colleagues’ skills or drive negatively impact their work standard | .036 | -.21 | .279 | .001 |
| j. Rush examinations due to time pressure | .154 | -.117 | .455 | .013 |
| l. Pace letting down the team | -.226 | -.375 | -.064 | .055 |
| m. Worry about amount of jobs team has each day | -.034 | -.252 | .192 | .001 |
| Years of service in Forensic Services Group | -.197 | -.395 | .014 | .041 |
| Gender | -.144 | -.292 | .015 | .026 |

*Note.* CI = Confidence interval; LB = Lower boundary; UB = Upper boundary.

TABLE S14 Regression results for forensic staff specific demands predicting job satisfaction (R^2^ = .38).

|  | **β** | **95% CI (LB)** | **95% CI (UB)** | ***f*^2^** |
| --- | --- | --- | --- | --- |
| a. Admin obligations overwhelm | -.235 | -.416 | -.052 | .052 |
| b. Hours of work impact work life balance | -.002 | -.166 | .157 | .000 |
| e. Doubting own thoroughness in investigation | -.356 | -.627 | -.036 | .058 |
| f. Wondering about missing something in examination | .175 | -.128 | .470 | .017 |
| g. Concerned colleagues’ skills or drive negatively impact their work standard | -.176 | -.393 | .069 | .026 |
| j. Rush examinations due to time pressure | -.144 | -.408 | .138 | .014 |
| l. Pace letting down the team | -.129 | -.295 | .024 | .021 |
| m. Worry about amount of jobs team has each day | .069 | -.093 | .239 | .006 |
| Years of service in Forensic Services Group | -.116 | -.264 | .037 | .017 |
| Gender | -.056 | -.207 | .107 | .004 |

*Note.* CI = Confidence interval; LB = Lower boundary; UB = Upper boundary.

TABLE S15 Regression results for forensic staff specific demands predicting perceived physical health (R^2^ = .14).

|  | **β** | **95% CI (LB)** | **95% CI (UB)** | ***f*^2^** |
| --- | --- | --- | --- | --- |
| a. Admin obligations overwhelm | -.034 | -.034 | -.034 | -.034 |
| b. Hours of work impact work life balance | .034 | .034 | .034 | .034 |
| e. Doubting own thoroughness in investigation | -.054 | -.054 | -.054 | -.054 |
| f. Wondering about missing something in examination | .026 | .026 | .026 | .026 |
| g. Concerned colleagues’ skills or drive negatively impact their work standard | -.109 | -.109 | -.109 | -.109 |
| j. Rush examinations due to time pressure | -.026 | -.026 | -.026 | -.026 |
| l. Pace letting down the team | -.047 | -.047 | -.047 | -.047 |
| m. Worry about amount of jobs team has each day | -.149 | -.149 | -.149 | -.149 |
| Years of service in Forensic Services Group | -.122 | -.122 | -.122 | -.122 |
| Gender | -.163 | -.163 | -.163 | -.163 |

*Note.* CI = Confidence interval; LB = Lower boundary; UB = Upper boundary.

TABLE S16 Regression results for resources predicting positive stress-related outcomes.

|  | **β** | **95% CI (LB)** | **95% CI (UB)** | ***f*^2^** |
| --- | --- | --- | --- | --- |
| **Work engagement (*R*^2^ = .29)** |  |  |  |  |
| Psychosocial safety climate - organisation | .158 | -.067 | .398 | .023 |
| Psychosocial safety climate - overviewing officer | .106 | -.151 | .375 | .008 |
| Supervisor support | .256 | .021 | .478 | .063 |
| Peer support | .133 | -.155 | .388 | .021 |
| Family support | .034 | -.199 | .289 | .001 |
| Years of service in forensic services | -.037 | -.169 | .109 | .002 |
| Gender | -.135 | -.294 | .027 | .028 |
| **Work performance (*R*^2^ = .17)** |  |  |  |  |
| Psychosocial safety climate - organisation | .232 | .008 | .435 | .045 |
| Psychosocial safety climate - overviewing officer | .113 | -.151 | .348 | .008 |
| Supervisor support | .098 | -.096 | .280 | .008 |
| Years of service in forensic services | -.056 | -.235 | .120 | .003 |
| Gender | -.102 | -.265 | .062 | .012 |
| **Job satisfaction (*R*^2^ = .40)** |  |  |  |  |
| Psychosocial safety climate - organisation | .295 | .123 | .449 | .099 |
| Psychosocial safety climate - overviewing officer | .101 | -.132 | .313 | .009 |
| Supervisor support | .244 | .020 | .441 | .063 |
| Peer support | .156 | -.085 | .417 | .033 |
| Family support | .061 | -.155 | .283 | .003 |
| Years of service in Forensic Services Group | -.036 | -.164 | .109 | .003 |
| Gender | -.086 | -.228 | .062 | .012 |
| **Perceived physical health (*R*^2^ = .17)** |  |  |  |  |
| Psychosocial safety climate - organisation | .227 | -.049 | .508 | .038 |
| Psychosocial safety climate - overviewing officer | -.077 | -.341 | .180 | .004 |
| Peer support | .236 | .044 | .412 | .050 |
| Years of service in Forensic Services Group | .029 | -.179 | .266 | .001 |
| Gender | -.123 | -.290 | .062 | .018 |

*Note.* CI = Confidence interval; LB = Lower boundary; UB = Upper boundary.

TABLE S17 Regression results for resources predicting negative stress-related outcomes.

|  | **β** | **95% CI (LB)** | **95% CI (UB)** | ***f*^2^** |
| --- | --- | --- | --- | --- |
| **Psychological distress (*R*^2^ = .24)** |  |  |  |  |
| Psychosocial safety climate - organisation | -.087 | -.308 | .135 | .008 |
| Psychosocial safety climate - overviewing officer | -.099 | -.328 | .164 | .008 |
| Supervisor support | -.235 | -.430 | -.030 | .046 |
| Peer support | -.031 | -.453 | .297 | .000 |
| Family support | -.148 | -.427 | .153 | .024 |
| Years of service in Forensic Services Group | .098 | -.039 | .222 | .013 |
| Gender | .061 | -.101 | .199 | .006 |
| **Vicarious trauma (*R*^2^ = .29)** |  |  |  |  |
| Psychosocial safety climate - organisation | -.194 | -.402 | .044 | .035 |
| Psychosocial safety climate - overviewing officer | .065 | -.164 | .314 | .003 |
| Supervisor support | -.327 | -.495 | -.132 | .103 |
| Peer support | -.047 | -.260 | .177 | .002 |
| Family support | -.171 | -.414 | .039 | .028 |
| Years of service in Forensic Services Group | .123 | -.063 | .304 | .023 |
| Gender | .102 | -.055 | .251 | .015 |
| **Burnout/exhaustion (*R*^2^ = .35)** |  |  |  |  |
| Psychosocial safety climate - organisation | -.067 | -.280 | .162 | .004 |
| Psychosocial safety climate - overviewing officer | .005 | -.235 | .239 | .000 |
| Supervisor support | -.204 | -.398 | -.007 | .044 |
| Peer support | -.282 | -.471 | -.076 | .089 |
| Family support | -.177 | -.390 | .047 | .034 |
| Years of service in Forensic Services Group | .143 | -.033 | .300 | .031 |
| Gender | .116 | -.031 | .273 | .022 |
| **Turnover intentions (*R*^2^ = .26)** |  |  |  |  |
| Psychosocial safety climate - organisation | -.213 | -.461 | .036 | .041 |
| Psychosocial safety climate - overviewing officer | -.281 | -.519 | -.012 | .056 |
| Supervisor support | -.130 | -.336 | .083 | .013 |
| Peer support | -.021 | -.218 | .197 | .000 |
| Years of service in Forensic Services Group | .002 | -.197 | .196 | .000 |
| Gender | -.018 | -.178 | .138 | .001 |

*Note.* CI = Confidence interval; LB = Lower boundary; UB = Upper boundary.
